# A warm welcome for alternative CO_2_ fixation pathways in microbial biotechnology

**DOI:** 10.1111/1751-7915.12456

**Published:** 2016-11-22

**Authors:** Nico J. Claassens

**Affiliations:** ^1^ Laboratory of MicrobiologyWageningen UniversityStippeneng 46708 WEWageningenThe Netherlands

**Figure 1:**
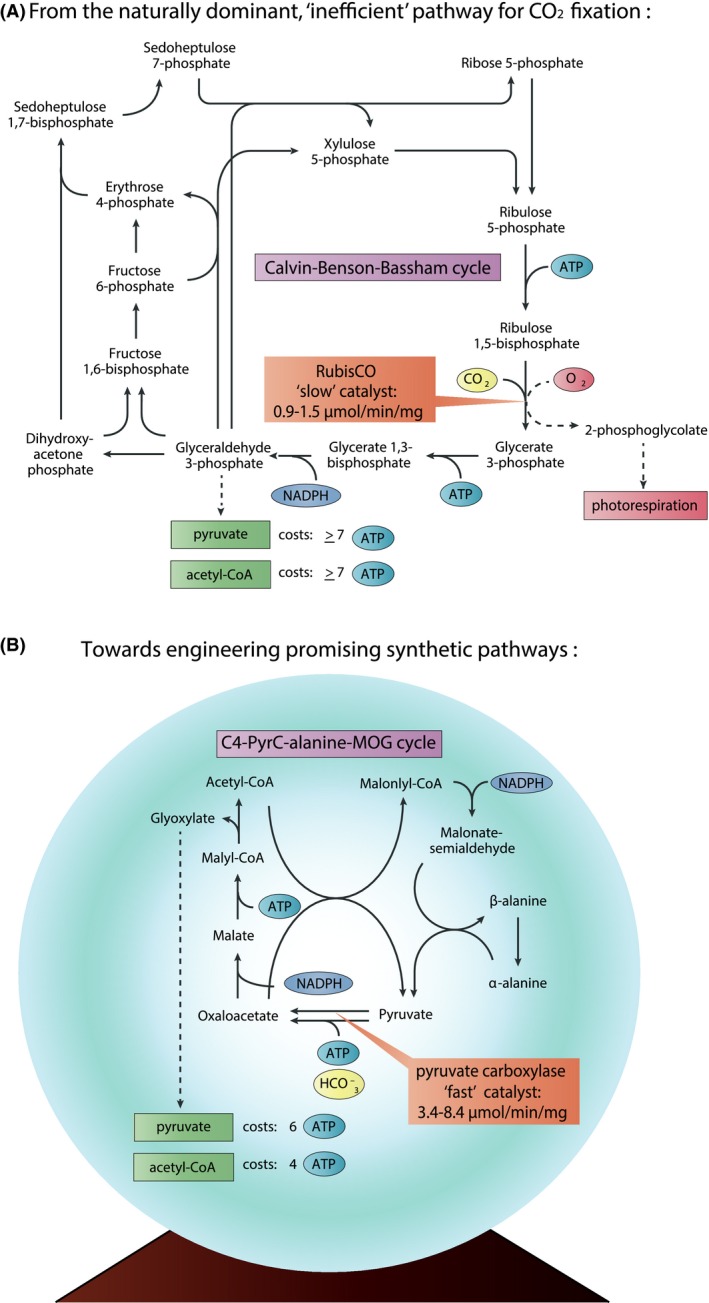
(A) The ‘inefficient’ Calvin–Benson–Bassham cycle with the ‘slow’ carboxylase RubisCO as key enzyme, the cycle has a consumption of seven ATP per pyruvate or acetyl‐CoA produced, which does not include additional costs for photorespiration in aerobic conditions (Bar‐Even *et al*., 2010). (B) An example of a proposed, promising synthetic MOG‐pathway, based on the ‘faster’ carboxylase pyruvate carboxylase (Bar‐Even *et al*., 2010), estimates for its ATP consumption are based on assimilation via the bacterial glycerate pathway for pyruvate and the reverse glyoxylate shunt for acetyl‐CoA. Catalytic turnover rates indicated for both carboxylases are based on ambient CO_2_ concentrations (Bar‐Even *et al*., 2010).

Biological CO_2_ fixation is a crucial process carried out by plants and a number of microorganisms, which can be harnessed for both agriculture and sustainable, bio‐based production of fuels and chemicals. Fixation of CO_2_ by plants enables the production of food, feed, fuels and chemicals. Additionally, fixation of CO_2_ by autotrophic microorganisms such as cyanobacteria and microalgae can be employed for converting CO_2_ into value‐added products, such as commodity chemicals or fuels. However, a major challenge to fully realize sustainable autotrophic production of chemicals and fuels is the low growth rate, productivity and energy conversion efficiency of autotrophs. Excluding the energy loss photoautotrophs experience due to inefficient light‐harvesting (Blankenship *et al*., 2011), another major energy loss occurs during the fixation of CO_2_ in both photoautotrophs and chemolithoautotrophs (Zhu *et al*., 2010). The large majority of autotrophs employ the relatively inefficient Calvin–Benson–Bassham cycle for CO_2_ fixation (Fig. 1A). After having a dominant role in nature for billions of years, in the coming years I expect synthetic biologists will replace the Calvin cycle in biotechnology with potentially better and more efficient alternatives.

But what is the limiting factor of the Calvin cycle? The main issue is the key enzyme ribulose‐bisphosphate carboxylase/oxygenase (RubisCO). RubisCO has a low catalytic rate, and therefore is generally expressed in high levels, which requires a considerable amount of cellular resources. This resource demand comes on top of the existing high ATP‐demand of the Calvin cycle required to produce common metabolic precursors such as acetyl‐CoA and pyruvate (Claassens *et al*., 2016). Furthermore, RubisCO has a major wasteful side‐activity with O_2_ in atmospheric conditions. This side‐activity results in the formation of 2‐phoshpoglycoylate that has to be re‐assimilated to the Calvin cycle through photorespiration pathways. These photorespiration pathways partly counteract CO_2_ fixation by releasing some CO_2_ and typically add ~40–50% extra NADPH and ATP to the costs of CO_2_ fixation (Bar‐Even *et al*., 2010).

In recent years, many ideas have been put forward and attempts have been made to increase the efficiency of the Calvin cycle including; improving catalytic properties of RubisCO by protein engineering (e.g. Parikh *et al*., 2006; Durão *et al*., 2015), introducing more efficient photorespiration pathways (e.g. Shih *et al*., 2014) and introducing CO_2_ concentrating mechanisms to increase the carboxylating activity of RubisCO (e.g. Bonacci *et al*., 2012; Kamennaya *et al*., 2015). Some of these attempts have slightly improved the performance of autotrophs employing the Calvin cycle. However, apart from fixing such inefficiencies of the Calvin cycle, future research should also seriously address the options to completely replace both the Calvin cycle and accompanying enzyme RubisCO by potentially more efficient alternatives.

Fortunately, nature has evolved many carboxylases with more promising properties than RubisCO and more attractive novel carboxylases may be created by protein engineering (Erb, 2011). Some examples of attractive natural carboxylases are employed in alternative natural carbon fixation pathways, thus far five alternative autotrophic CO_2_ fixation pathways have been discovered (Berg, 2011). Moreover, attractive carboxylases can be embedded in synthetic CO_2_ fixation pathways, which have been extensively explored by computational analyses (e.g. Bar‐Even *et al*., 2010; Volpers *et al*., 2016). Several of the alternative natural and synthetic CO_2_ fixation pathways have lower ATP costs than the Calvin cycle, however, some trade‐offs exist. For example, the natural Wood–Ljungdahl pathway and the natural reductive tricarboxylic acid cycle have very low ATP costs. However, these pathways can have major drawbacks, as these pathways are mainly limited to anaerobic settings, due to oxygen‐sensitive enzymes, and they require high CO_2_ concentrations to be thermodynamically feasible (Berg, 2011). Alternative to natural pathways, computational analysis identified several oxygen‐tolerant synthetic pathways that while taking multiple criteria into account, are predicted to have an overall better performance than the Calvin cycle. As an example, a promising group of synthetic pathways are the malonyl‐CoA–oxaloacetate–glyoxylate (MOG) pathways (Fig. 1B) (Bar‐Even *et al*., 2010). These pathways include the catalytically fast phosphoenol pyruvate carboxylase or pyruvate carboxylase and are expected to have fast overall pathway kinetics. In addition, these MOG pathways involve only oxygen‐tolerant enzymes and have predicted lower ATP costs than the Calvin cycle. In addition, synthetic pathways based on crotonyl‐CoA carboxylases, the fastest known carboxylases, may be highly promising to apply in synthetic CO_2_ fixation pathways (Erb *et al*., 2009).

Attractive natural pathways are only found in a few, limitedly characterized microorganisms, which are generally less suitable for biotechnology. The alternative synthetic pathways are not (yet) known to occur in natural microorganisms. Therefore, to realize the potential of alternative CO_2_ fixation pathways they should be introduced into suitable microbial hosts via metabolic engineering. Hereto, in recent years a few attempts have been made, such as the introduction of the complete natural 3‐hydroxypropionate bi‐cycle in *Escherichia coli* by the Silver laboratory, which did not yet yield a completely functional pathway (Mattozzi *et al*., 2013). In the Adams laboratory, a section of the natural 3‐hydroxypropionate/4‐hydroxybutyrate cycle was successfully introduced in the heterotrophic thermophile *Pyrococcus furiosus*; however, this short section is insufficient to support complete autotrophic growth (Keller *et al*., 2013).

These partly successful examples demonstrate that introduction of a full functional CO_2_ fixation pathway is a major challenge. To allow for more efficient autotrophic growth, the engineered CO_2_ fixation pathway has to become a key functional component of central metabolism and subsequently the alternative pathways should be properly integrated with native metabolism. This will involve reregulation of native metabolism, for which a rational engineering approach will probably be insufficient. However, the power of adaptive laboratory evolution will be very helpful to optimize pathways and re‐regulate native metabolism. CO_2_ fixation pathways are a perfect case for adaptive laboratory evolution, as their functionality can often be easily coupled to a growth phenotype. This has been exemplified very recently by the Milo laboratory for the engineering of the Calvin cycle in *E. coli*, for which they coupled pathway activity to growth of *E. coli*. After about 150 generations in a chemostat, laboratory evolution led to the first completely functional, engineered CO_2_ pathway in a heterotroph (Antonovsky *et al*., 2016). Even though this major achievement was shown for the ‘inefficient’ Calvin cycle, which requires only three heterologous genes in *E. coli*, this approach opens up the door for future engineering of more promising, extensive CO_2_ fixation pathways in heterologous hosts.

A general challenge for engineering CO_2_ fixation pathways in *E. coli* and other model heterotrophs is the lack of autotrophic energy systems to regenerate ATP and reducing equivalents such as NADPH. Therefore, autotrophs already harbouring these systems for ATP and NADPH regeneration are promising hosts for engineering alternative CO_2_ fixation pathways. Compared to model heterotrophs, such as *E. coli* and yeast, the available genetic toolboxes for autotrophs are more limited. However, given the ongoing tool development in some biotechnologically relevant autotrophs, these strains should be considered for engineering CO_2_ fixation pathways (Claassens *et al*., 2016). Promising autotrophs include photoautotrophs, such as the cyanobacterium *Synechocystis* PCC6803 and anoxygenic phototroph *Rhodobacter sphaeroides*, and some chemolithoautotrophs, such as *Cupriavidus necator* (formerly *Ralstonia eutropha*), the latter uses hydrogen and CO_2_ for autotrophic growth. All mentioned promising biotechnological autotrophs contain a native Calvin cycle, which could be replaced by potentially more efficient pathways. By knocking out RubisCO in these autotrophs, the activity of introduced alternative CO_2_ fixation pathways can be coupled to their autotrophic growth, providing a proper basis for adaptive laboratory evolution.

Some laboratories are already undertaking approaches to engineer and evolve synthetic CO_2_ fixation in both microbial heterotrophs and autotrophs. In addition, some laboratories are testing promising synthetic CO_2_ fixation pathways *in vitro*, by combining purified enzymes for sections or complete pathways in a tube. These *in vitro* approaches will be helpful to test pathway functionality, especially for synthetic pathways that involve insufficiently characterized natural enzymes or engineered enzymes. For example, issues regarding undesired side‐activities can be identified more easily *in vitro*. Recently, the Erb laboratory successfully used the *in vitro* approach for the first‐demonstrated synthetic pathway: the CETCH‐cycle, which involves the kinetically fast carboxylase crotonyl‐CoA carboxylase (Schwander and Erb, 2016). However, after *in vitro* pathway debugging and a proof‐of‐principle for functionality, subsequent *in vivo* engineering and testing should be the key approach for engineering novel pathways. Only an *in vitro* approach will allow for proper assessment of the performance of alternative pathways in a physiological context and the realization of more efficient autotrophic cell factories.

I expect that engineering alternative CO_2_ fixation pathways in autotrophs will pave the road for more efficient autotrophic production strains. In addition, lessons learnt by engineering synthetic CO_2_ fixation will be important for the even larger challenge of engineering improved, synthetic CO_2_ fixation pathways in plants (Ort *et al*., 2015). Endeavours in this direction may enable realization of the large promise of more efficient agricultural crops. However, these undertakings will be even more challenging than for microbes, given the more limited genetic tools and longer generation times, hampering evolutionary approaches in plants. Moreover, one should not underestimate the large societal resistance against genetically modified plants. Concluding, for autotrophic, biotechnologically relevant microbes that got ‘stuck in evolution’ with the ‘inefficient’ Calvin cycle, we now have knowledge and tools to aim to replace this pathway by more efficient alternatives. It is now up to synthetic biologists to pick up this exciting and promising challenge to realize more efficient autotrophic production platforms for a sustainable bio‐economy.
